# Application of a High-Content Screening Assay Utilizing Primary Human Lung Fibroblasts to Identify Antifibrotic Drugs for Rapid Repurposing in COVID-19 Patients

**DOI:** 10.1177/24725552211019405

**Published:** 2021-06-02

**Authors:** John A. Marwick, Richard J. R. Elliott, James Longden, Ashraff Makda, Nik Hirani, Kevin Dhaliwal, John C. Dawson, Neil O. Carragher

**Affiliations:** 1 Cancer Research UK Edinburgh Centre, Institute of Genetics and Cancer, University of Edinburgh, Edinburgh, UK; 2 Centre for Inflammation Research, Queens Medical Research Institute, University of Edinburgh, Edinburgh, UK; 3 Center for Clinical Brain Sciences, Chancellors Building, University of Edinburgh, Edinburgh, UK

**Keywords:** lung fibrosis, COVID-19, extracellular matrix, high-content screening, phenotypic drug discovery

## Abstract

Lung imaging and autopsy reports among COVID-19 patients show elevated lung scarring (fibrosis). Early data from COVID-19 patients as well as previous studies from severe acute respiratory syndrome, Middle East respiratory syndrome, and other respiratory disorders show that the extent of lung fibrosis is associated with a higher mortality, prolonged ventilator dependence, and poorer long-term health prognosis. Current treatments to halt or reverse lung fibrosis are limited; thus, the rapid development of effective antifibrotic therapies is a major global medical need that will continue far beyond the current COVID-19 pandemic. Reproducible fibrosis screening assays with high signal-to-noise ratios and disease-relevant readouts such as extracellular matrix (ECM) deposition (the hallmark of fibrosis) are integral to any antifibrotic therapeutic development. Therefore, we have established an automated high-throughput and high-content primary screening assay measuring transforming growth factor-β (TGFβ)-induced ECM deposition from primary human lung fibroblasts in a 384-well format. This assay combines longitudinal live cell imaging with multiparametric high-content analysis of ECM deposition. Using this assay, we have screened a library of 2743 small molecules representing approved drugs and late-stage clinical candidates. Confirmed hits were subsequently profiled through a suite of secondary lung fibroblast phenotypic screening assays quantifying cell differentiation, proliferation, migration, and apoptosis. In silico target prediction and pathway network analysis were applied to the confirmed hits. We anticipate this suite of assays and data analysis tools will aid the identification of new treatments to mitigate against lung fibrosis associated with COVID-19 and other fibrotic diseases.

## Introduction

There is increasing evidence that the aberrant immune response in the lungs instigated by the severe acute respiratory syndrome coronavirus 2 (SARS-COV-2) leads to lung scarring and fibrosis.^1^ This ranges from fibrosis associated with organizing pneumonia to more severe lung injury, with widespread fibrotic change.^2^ Lung fibrosis is observed in almost all fatal cases of COVID-19.^3^ Furthermore, there is a high rate of interstitial lung function abnormalities in COVID-19 patients discharged from the hospital, with 47% (of 110 patients studied) showing impaired gas transfer and 25% showing reduced total lung capacity.^4^ The degree of this lung function compromise may have an important impact on the long-term recovery and prognosis of COVID-19 survivors. Data from similar conditions including SARS, Middle East respiratory syndrome (MERS), and subsets of acute respiratory distress syndrome (ARDS) show that patients all develop lung fibrosis, with greater fibrosis associated with prolonged ventilator dependence, higher mortality rate, and poorer prognosis in long-term health-related quality of life.^5–8^

The precise cellular and molecular mechanisms that underpin lung fibrosis in diseases like idiopathic lung fibrosis (IPF) remain unclear. This in turn hinders the design of appropriate preclinical models with which to investigate the diseases for drug discovery and the identification of the most effective therapeutic targets. However, what is clear from pathology studies in lung fibrosis patients is the common occurrence of fibroproliferation with associated aberrant deposition of extracellular matrix (ECM) within the lung, leading to impaired gas exchange and lung function that can be progressive and terminal.^9^ Therefore, targeting fibroblast “fibroproliferation” and associated ECM deposition as adjunctive interventions to immunosuppressive therapies represents a logical therapeutic strategy for both delaying disease onset and, potentially, reversal of disease pathology.

There are only two drugs approved for specifically treating IPF—pirfenidone and nintedanib. These have proven partially effective at controlling lung diseases such as IPF, and studies are ongoing as to their effectiveness toward the fibroproliferative response in COVID-19.^1^ Due to so few current antifibrotic therapies available and the substantial risk of increased incidence of lung fibrosis post-COVID-19 infection, there is a critical need for further investment in improved drug discovery screening cascades that incorporate more relevant and informative laboratory models of lung fibrosis. The application of target-agnostic phenotypic assays with multiple readouts is well placed to provide improved hit rates for potential antifibrotic therapies via the empirical identification of novel drug candidates or repurposed drugs that target distinct pathway mechanisms.

To help innovate the identification of novel targets, new chemical starting points, and potential drug repurposing candidates for tackling lung fibrosis, we established an automated high-throughput and high-content assay measuring primary human lung fibroblast proliferation and ECM deposition. To rapidly repurpose drugs toward clinical trials for the treatment of patients with or recovering from COVID-19, we prioritized the screening of a library of 2743 small molecules predominantly composed of approved drugs and late-stage clinical candidates. Here, we describe the development and application of our primary high-content screen and a suite of image-based secondary lung phenotypic assays to mechanistically profile and triage those hits that may have greatest value in preventing, halting, or reversing lung fibrosis in patients with COVID-19. We identify the following six drugs, which demonstrate potent and reproducible activity in our lung phenotypic assays: RepSox, camptothecin, diphenylene-iodonium chloride, GSK-650394, niclosamide, and fenretinide. We further describe how combining multiparametric high-content image analysis, structural similarity ligand-based target prediction, and pathway network analysis deconvolutes the mechanism of action of our phenotypic hits at the target and pathway network level. We present full results from our screening assays and assay protocols to support the identification of new potential therapies to reduce aberrant pulmonary fibrosis in COVID-19 patients and other lung diseases.

## Materials and Methods

### Primary Human Lung Fibroblast Culture

Primary human lung fibroblasts from healthy donors (American Type Culture Collection [ATCC], Manassas, VA, PCS-201-013) were expanded by three passages (21 days in culture), followed by cryopreservation as assay-ready stocks. Fibroblast culture was performed using fibroblast growth medium-2 (FGM-2) BulletKit media (Lonza, CC-3132, Slough, UK) under standard tissue culture conditions (37 °C and 5% CO_2_). Fibroblasts were harvested by washing twice with Dulbecco’s phosphate-buffered saline (DPBS) (Thermo Fisher Scientific, Waltham, MA) incubated with TrypLE Express (Thermo Fisher Scientfic) at 37 °C for 4 min, diluted using FGM-2 BulletKit media and centrifuged for 5 min at 200*g*. The cells were resuspended in either FGM-2 BulletKit media for continued passage or cryopreservation, or Renal Epithelial Cell Basal Medium (ATCC, PCS-400-030) supplemented with Renal Epithelial Cell Growth Kit (ATCC, PCS-400-040) for high-content compound screening. Cell counts were performed using a NucleoCounter NC-100 with NucleoCassettes per the manufacturer’s protocol (Chemometec, Allerod, Denmark).

### High-Content ECM Deposition Assay

Fibroblasts were resuspended in treatment media at 80,000 cells/mL and then seeded into 384-well imaging plates (Greiner microclear, Greiner Bio-One GmbH, Kremsmunster, Austria) at 25 µL/well, with a further 25 µL of media with or without 10 ng/mL rhTGFβ (R&D Systems, Minneapolis, MN, 7754-BH/CF; final concentration, 5 ng/mL). The plates were given a pulse spin (200*g* for 1 s) and then placed in an incubator overnight. The following day, the compounds (1 µM) or DMSO control (0.1% v/v) were added using a BioMek liquid handling unit (Beckman Coulter, Brea, CA). Compound libraries included the Prestwick Chemical Library (Prestwick Chemical, Illkirch, France) and LOPAC1280 (Library of Pharmacologically Active Compounds; Merck, Darmstadt, Germany). The plates were then placed into the IncuCyte Live Cell Imaging System (Sartorius, Gottingen, Germany) and imaged every 3 h for 6 days.

ECM was stained using a protocol adapted from Holdsworth et al.^10^ Briefly, the cells were lysed using an ammonia-based lysis media (0.25 M NH_4_OH, 25 mM Tris) for 30 min at 37 °C, followed by washing three times with phosphate-buffered saline (PBS). The ECM was fixed using ice-cold methanol for 20 min at −20 °C, followed by washing three times with PBS. Assay plates were then blocked using 1% (v/v) goat serum (G9023, Thermo Fisher Scientific) in PBS for 45 min, followed by labeling with anti-collagen I (Millipore, Burlington, MA, AB745; 1 in 100), anti-collagen III (Millipore, AB747; 1 in 100), and anti-collagen IV (Ebioscience, San Diego, CA, 14-9871-82; Millipore; 1 in 100) antibodies diluted in blocking buffer for 1.5 h, followed by three washes with PBS. The cells were then incubated with Alexa Fluor 555 Goat anti-Rabbit IgG (Life Technologies, Carlsbad, CA, A21430; 1 in 400) and Alexa Fluor 647 Goat Anti-Mouse IgG (Life Technologies, A21236; 1 in 400) for 1.5 h, followed by washing three times with PBS. The cells were then labeled with Alexa Fluor 488 anti-fibronectin (Ebioscience, 53-9869-82; 1 in 400) for 1.5 h, followed by washing three times with PBS and stored in the dark at 4 °C.

### High-Content ECM Deposition Assay: Image Acquisition and Analysis

Plates were imaged on an ImageXpress high-content microscope (Molecular Devices, San Jose, CA) using a 10× objective capturing four fields of view per well. ECM deposition was measured using a custom analysis created in the Custom Module Editor (MetaXpress, Molecular Devices). Briefly, a mask for each fluorescent channel containing ECM was calculated by selecting a user-defined threshold. All three ECM component masks (collagen I+III, collagen IV, and fibronectin) were combined to form a “total ECM mask” against which the intensity of the fluorescent antibody labeling in each individual channel could be measured. Image-level data were aggregated using the SUM function, exported, and analyzed using HC StratoMineR software (Core Life Analytics, ‘s-Hertogenbosch, Netherlands). Briefly, data were normalized to percentage inhibition of transforming growth factor-β (TGFβ)-induced ECM deposition and scaled using a robust Z score. Hit compounds were selected using a Euclidean distance from the negative control with a *p* value cutoff of 0.05 (corrected using the false discovery rate).

### Primary Lung Fibroblast αSMA Expression and Proliferation Assay

Primary human lung fibroblasts were plated, TGFβ stimulated, and drug treated in 384-well plates as per the ECM deposition assay. An untreated plate was also cultured overnight, fixed, and labeled with HCS CellMask Green (Invitrogen, Themo Fisher Scientific, H32714) and Hoechst 33342 (Invitrogen, R37605) for a cell count at day 1. Plates were drug treated for 6 days and then fixed using 4% formaldehyde (Pierce, 28906) in PBS for 15 min at 37 °C, followed by three washes with PBS. Day 1 and drug-treated plates were permeabilized with 0.1% Triton X-100 in PBS (Thermo Fisher Scientific, 28313) for 10 min, followed by two washes with PBS, followed by blocking (10% goat serum, 1% bovine serum albumin [BSA], 0.3 M glycine in PBS with 0.1% Tween) for 45 min. The cells were then labeled with anti-α-smooth muscle actin (anti-αSMA) (Abcam, Cambridge, UK, ab7817; 1 in 200) diluted in 1% BSA in PBS/0.1% Tween) overnight. Plates were washed three times with PBS and labeled with Alexa Fluor 647 anti-mouse secondary antibody (Thermo Fisher Scientific, A-21236; 1 in 400) diluted in 1% BSA-PBS/0.1% Tween for 1.5 h. Plates were washed three times in PBS and labeled with HCS CellMask Green (1 in 20,000) and Hoechst 33342 (1 in 15) in PBS for 30 min and finally washed three times with PBS. The plates were then stored in the dark prior to image acquisition using a 10× objective on the ImageXpress (Molecular Devices).

The expression of αSMA was measured with a custom analysis using the Custom Module Editor (MetaXpress, Molecular Devices). Briefly, Hoechst 33342 labeling was used to create a nuclei mask identified with a user-defined threshold. The nuclei mask was then used to identify cytoplasmic fluorescence for each cell and to generate a mask for αSMA and HSC CellMask Green labeling with a user-defined threshold. Nuclei, αSMA, and HCS CellMask Green masks were combined to create a whole-cell mask that was used to measure the integrated intensity of each individual component.

### Scratch Wound Migration Assay

The scratch wound assay was performed and imaged using the IncuCyte Zoom Live Cell Imaging System and Scratch Wound analysis module per the manufacturer’s instructions (IncuCyte, Sartorius). Briefly, human lung fibroblasts were resuspended in treatment media with 5 ng/mL TGFβ at 150,000 cells/mL, and cells were seeded in 96-well plates at a final density of 15,000 cells/well (Corning, New York, NY) and incubated overnight. The following day, scratch wounds were performed using the IncuCyte WoundMaker as previously described.^11^ The media was then replaced to remove cell debris, compounds were added, and the plate was imaged every 2 h using the IncuCyte for 4 days. Wound closure was calculated using IncuCyte’s wound confluence analysis module for each image over time.

### Apoptosis Assay

Human lung fibroblasts were resuspended in treatment media with 5 ng/mL TGFβ at 40,000 per milliliter and then seeded into 384-well imaging plates (Greiner, 781091) at 25 µL/well. The plates were given a pulse spin (200*g* for 1 s) and then placed in an incubator overnight. The following day, the compounds or DMSO (0.1% v/v final concentration) control were added, followed by an apoptosis reporter (IncuCyte Caspase 3/7 Green; Sartorius, 4440; 5 µM), as previously described.^12^ The plates were then imaged every 2 h for 48 h in an IncuCyte Live Cell Imaging System. Apoptotic cells were identified using the IncuCyte’s analysis software to count the caspase 3/7-positive (green) cells in each image.

### Hit Confirmation Concentration Response and EC50 Determination

The 32 primary assay hit compounds were reordered from new commercial suppliers (MolPort, Riga, Latvia) and hit confirmation dose response plates were prepared as a 10-point semi-log concentration and added to the primary high-content ECM deposition assay at a final concentration of between 10 µM and 0.3 nM. ECM deposition was analyzed as described above, and EC50 values for compounds across each ECM channel were calculated by fitting the concentration response data to a standard four-parameter equation using Prism software (GraphPad, San Diego, CA). The top six potent compounds were also tested using a 10-point semi-log concentration range in the secondary lung fibroblast phenotypic profiling assays: αSMA expression, proliferation, apoptosis, and scratch wound migration.

### Structural Similarity Ligand-Based Target Predictions (SEA Search)

A structure-based in silico search of hit molecules was carried out using the publicly available, online resource Similarity Ensemble Approach (SEA) search (http://sea.bkslab.org).^13^ A SMILES string of each molecule (from Prestwick and LOPAC data files) was pasted into the SEA search fields. The SEA output includes a ranking of the proposed target/query interaction by probability and similarity score (Tanimoto coefficient). Significant predictions were further investigated via the ZINC database^14^ and links to other chemo-resources.

### Network Analysis

Network analysis was performed using Cytoscape v3.8.2.^15^ Protein targets of hit compounds were identified using the STITCH-STRING app (v1.6.0)^16^ to retrieve protein–compound and protein–protein interactions using a confidence score cutoff of 0.4 with up to 30 additional interactions. Functional analysis of hit compound–protein networks was analyzed using the ClueGO app v2.5.7^17^ showing only significant REACTOME pathways (*p* < 0.05). SEA search predictions were manually added to the networks.

## Results

### Assay Development: High-Content Primary Lung Fibroblast ECM Deposition Assay

**Figure 1.**
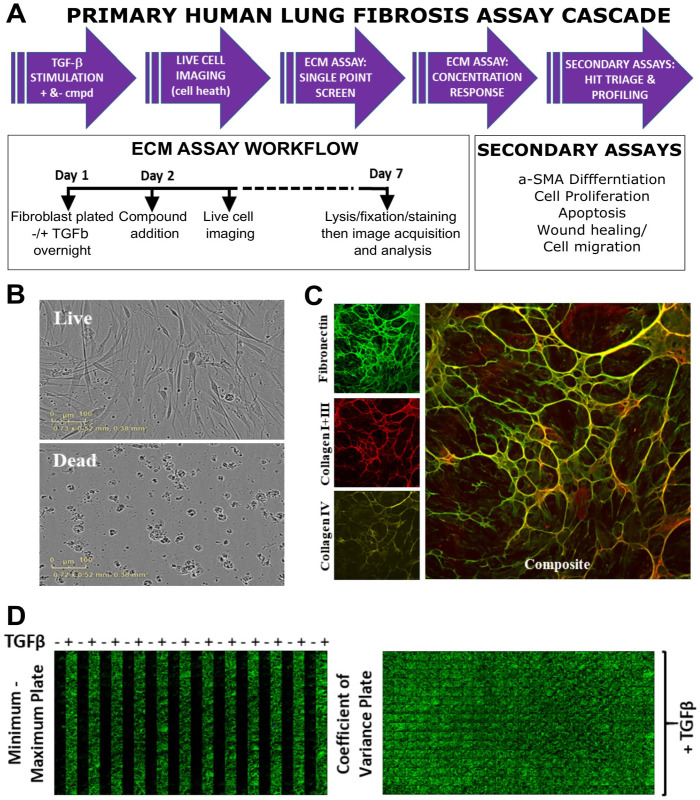
ECM and triage assay workflow. (**A**) Workflow for screening compounds through the ECM and triage assays. (**B**) Representative images from live cell imaging showing viable cells (top) and cell death (bottom) induced by compound cytotoxicity. (**C**) Representative high-content images of fibronectin, collagen I+III, and collagen IV staining with composite image. (**D**) Representative full-plate montage images showing fibronectin staining with or without TGFβ (5 ng/mL) to assess the assay window between minimum and maximum ECM deposition (left) and TGFβ (5 ng/mL)-induced fibronectin staining across a full 384-well plate for coefficient of variance assessment (right).

Primary human lung fibroblasts become increasingly sensitive to cryopreservation and senescence after repeated subculture, which can affect assay reproducibility across sequential cell culture passages. To mitigate this for our suite of primary human lung pro-fibrotic assays, original fibroblast stocks (ATCC, PCS-201-013) were rapidly expanded over three passages (no more than 21 days in culture in total) before being cryopreserved as assay-ready stocks. All assays were performed directly from these stocks with no further subpassaging between assays, which provided a foundation of cell phenotype reproducibility from which the assays could be performed. The density of primary human lung fibroblasts in culture greatly affects their phenotypic responses and particularly their susceptibility to compound toxicity. Accurate cell counting is therefore also an issue with primary human fibroblasts assays as they form tight clumps in suspension. This was mitigated by using a NucleoCounter (Chemometec), which performs a cytoplasmic lysis before counting stained nuclei, allowing consistent seeding densities between assays and across screens. For our 384-well high-content ECM deposition assays, a seeding density of 2000 cells/well was determined to be optimal. We have established and describe our full lung pro-fibrotic assay cascade, including the primary screen of human lung fibroblast ECM deposition with parallel live cell imaging, secondary phenotypic assays, and in silico hit target prediction and pathway network analysis (**Fig. 1A**).

As ECM deposition is a cell-free endpoint, it is difficult to triage compound hits in terms of compound cytotoxicity. If there is no or a significant reduction in ECM, without cell information it would be impossible to determine if this was due to specific inhibition of ECM synthesis, secretion and deposition, or simply significant lung fibroblast cytotoxicity of the compound. Therefore, to identify cytotoxic compounds, all assay plates were monitored by live cell imaging every 3 hours using an IncuCyte Zoom microscope during the course of the assay and immediately prior to cell lysis and ECM deposition labeling. Live cell imaging of assay plates therefore provided longitudinal phenotypic assessment of cell health following compound treatment for direct correlation with ECM deposition and the ability to triage the clearly cytotoxic compound hits from the screen (**Fig. 1B**).

Fibroblasts do not readily deposit ECM when cultured in proprietary fibroblast growth media, which is formulated to maintain a proliferative state, rather than allow a myofibroblast-like terminal differentiation that is necessary for ECM deposition. Therefore, all assays were run using renal proximal tube epithelial cell (RPTEC) media which Holdsworth et al.^10^ previously showed to enable reproducible mature ECM deposition from lung fibroblasts upon stimulation with TGFβ. Following immunolabeling of the ECM components (fibronectin and collagen types I, III, and IV) (**Fig. 1C**), imaging of the ECM was performed using an ImageXpress high-content imaging system and images analyzed using a custom module in MetaXpress software. We used untreated cells (no TGFβ) as a negative control to compare the ECM deposition induced by 5 ng/mL TGFβ. Following a series of time course and concentration–response curves, an optimal concentration of 5 ng/mL TGFβ for 7 days was identified as the optimal assay condition to induce reproducible ECM deposition. Intraplate coefficient of variance values for fibronectin (12.9%), collagen I+III (14.8%), and collagen IV (16.6%) demonstrate good assay reproducibility following TGFβ stimulation of ECM deposition (**Fig. 1D**).

### High-Content Primary Lung Fibroblast ECM Deposition Assay

#### Small-Molecule High-Content Screen

**Figure 2.**
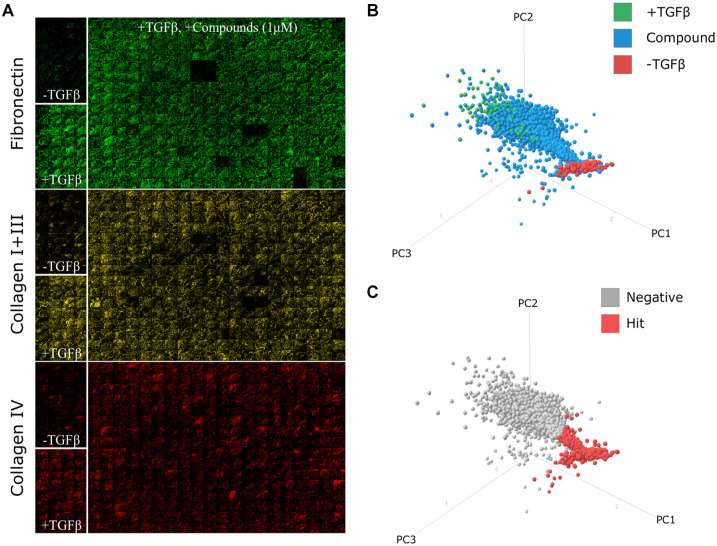
Primary human lung fibroblast ECM deposition high-content screen. Primary human lung fibroblasts were treated with TGFβ (5 ng/mL) over 7 days in the presence of compounds (1 µM). Plates were denuded of cells and ECM components immunolabeled and imaged on an ImageXpress microscope. (**A**) Exemplar images of ECM deposition. (**B**) Principal component (PC) analysis of the quantification of ECM deposition in response to compound treatment. (**C**) Hit classification using a Euclidean distance from the negative control with a *p* value cutoff of 0.05 (corrected using the false discovery rate).

**Figure 3.**
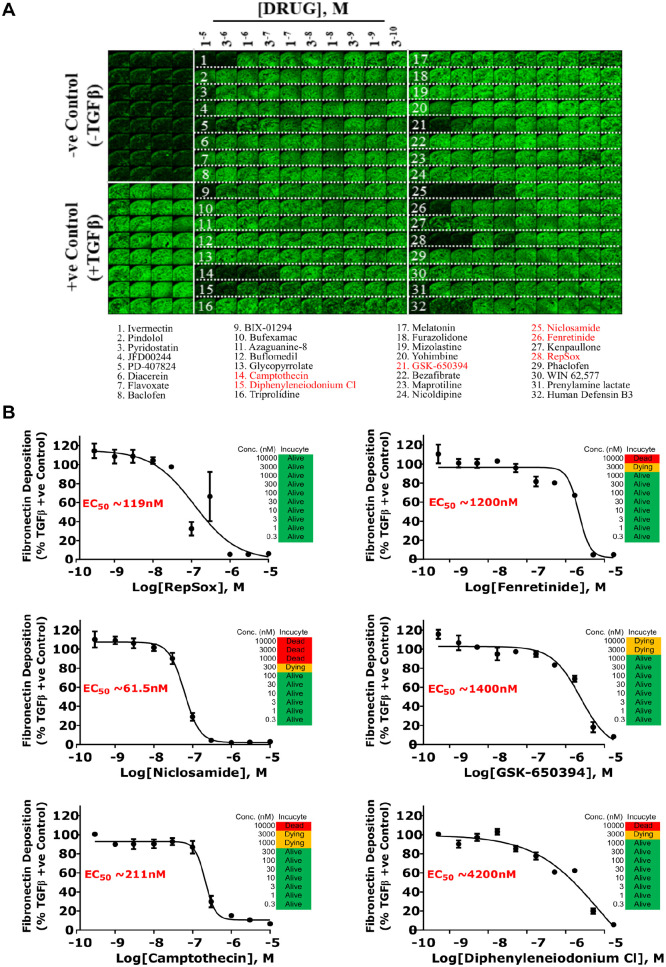
Selected hit compound 10-point concentration response in ECM assay. (**A**) Full-plate montage of fibronectin staining showing 10-point concentration response of 32 compound hits selected from the primary screen. Compound names listed, with the most active compounds highlighted in red. (**B**) Concentration responses of fibronectin deposition and cytotoxicity for the six compounds that reduced ECM deposition in a concentrated-related manner (see **Suppl. Figs. S1–S3** for collagen I+III and IV data). Conc., concentration.

A compound library of 2743 small molecules was screened at a single concentration of 1 µM in the ECM deposition assay. This library comprised the commercially available Prestwick Chemical Library of 1280 mostly off-patent Food and Drug Administration- and European Medicines Agency-approved compounds, the LOPAC library (1280 compounds), the Enzo SCREEN-WELL chemogenomic library of 176 epigenetic protease and kinase inhibitors, and a small selection of compounds of interest from collaborators. In brief, primary human lung fibroblasts were automatically dispensed into a 384-well plate in the presence of the inflammatory stimulus TGFβ (5 ng/mL) to induce ECM production. Twenty-four hours after cell plating, compounds were automatically dispensed at a final concentration of 1 µM using a Biomek FX. Following compound addition, cell assay plates were monitored in the IncuCyte Live Cell Imaging System, prior to cell lysis, fixation, and ECM labeling after 6 days of compound treatment (**Fig. 1**) (see Materials and Methods for further details). Control wells of lung fibroblasts ± TGFβ stimulation (32 wells of each) were included on each assay plate (**Fig. 2A**). Images of ECM deposition across assay plates were automatically acquired on an ImageXpress high-content platform (Molecular Devices) integrated with Peak Analysis and Automation plate handling robotics (PAA, Farnborough, UK). The fluorescent intensity of the ECM deposited in each image was measured and data were analyzed using the HC StratoMineR analysis software. Briefly, data were normalized on a per plate basis as percentage inhibition of TGFβ-induced ECM deposition. This analysis gave an overall hit rate of 10.6% (292 compounds) (**Fig. 2B**). The high hit rate is partly due to cytotoxic compounds, which simply reduced the ECM endpoint by killing the cells rather than inducing a specific phenotypic change. To filter out overtly cytotoxic compounds from the primary hit selection, cell confluence was calculated from the IncuCyte live cell images collected at day 6 of the assay. The live cell images from active compounds with less than 50% confluence at day 6 were then visually assessed to confirm cytotoxicity and then removed from the hit list. In total, 32 hit compounds from the ECM high-content analysis with no evidence of overt cytotoxicity (**Fig. 3A**) were selected for hit confirmation studies.

#### Hit Confirmation Assay: 10-Point Concentration Response

The 32 compounds prioritized from the primary screen were reordered from different commercial compound suppliers and retested across a 10-point semi-log concentration range using the high-content ECM deposition assay (**Fig. 3**). Here, 14 of the 32 compounds demonstrated some activity upon reducing ECM deposition in the assay at the top two concentrations of 10 and 3 µM (**Fig. 3A**). The following six compounds demonstrated the most potent concentration-dependent activity in reducing ECM deposition: RepSox, camptothecin, diphenylene-iodonium chloride, GSK-650394, niclosamide, and fenretinide. EC50 values for the reduction of ECM deposition were calculated to be in the range of 38.6 nM– 4.2 µM (**Fig. 3B and Suppl. Figs. S1–S3**). The results of compound activity on deposition of collagen types I, III, and IV and representative live cell images 6 days following compound treatments are shown in **Supplemental Figures S1–S3**. Validated hit compounds were assessed for cytotoxicity by reviewing the live cell images at day 6 post-compound addition, and each concentration was annotated as to whether cytotoxic activity was observed (**Fig. 3B and Suppl. Figs. S1–S3**). RepSox is a selective and potent inhibitor of the TGFβ type 1 receptor (TGFβRI) ALK5 and, as expected, inhibited TGFβ-induced deposition of ECM in this assay (**Fig. 3B and Suppl. Fig. S1**) (collagen I+III, EC50 = 38.6 nM; collagen IV, EC50 = 58.7 nM; fibronectin, EC50 = 119 nM). While TGFβ remains an important target in fibrosis, RepSox was also judged to be a useful assay control for future phenotypic screens, as it not only blocked the deposition of ECM but also did so with no effect on cell death. RepSox was also taken forward as a comparator for the other more novel hit compounds in secondary assays.

### Secondary Hit Triage and Compound Profiling Assays

The six most potent compounds confirmed by the 10-point semi-log concentration response studies were taken forward into four further secondary assays to assess the activity of each compound on key areas of fibroblast biology: (1) fibroblast to myofibroblast differentiation using the expression of αSMA as a marker, (2) primary lung fibroblast proliferation (nuclear count), (3) apoptosis of lung fibroblasts using a caspase 3/7 biosensor, and (4) lung fibroblast wound healing using a scratch wound migration assay.

#### αSMA Expression and Cell Proliferation

**Figure 4.**
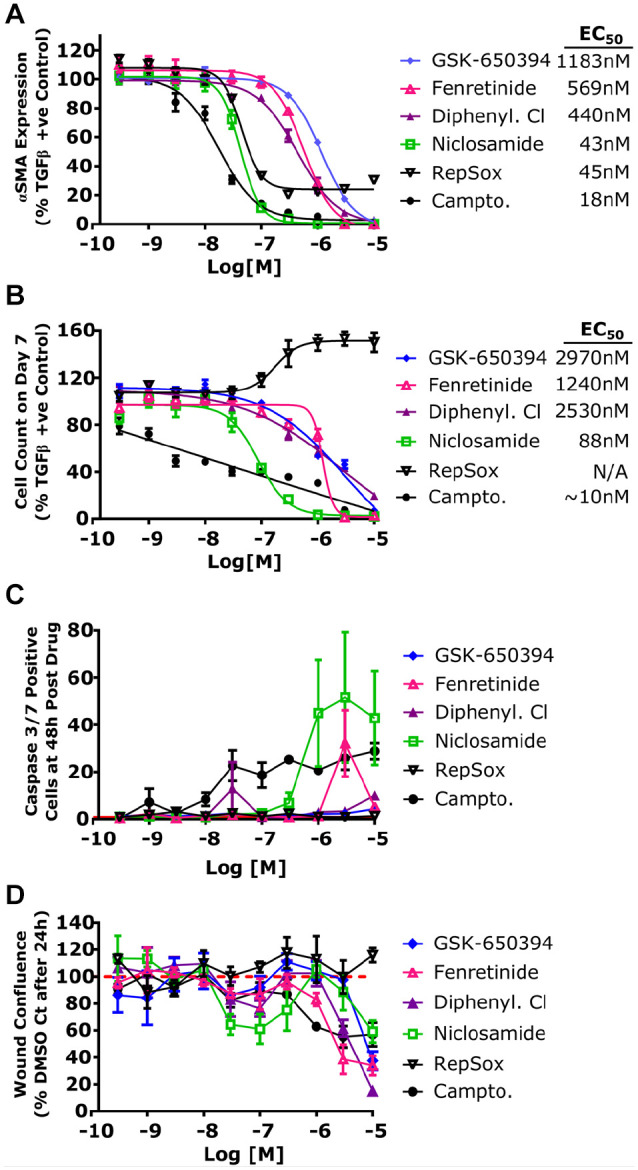
Secondary hit triage assays. (**A**) Concentration response of the six hit compounds on αSMA expression measured as a percentage of TGFβ (DMSO) positive control (see **Suppl. Fig. S4** for representative high-content image). (**B**) Concentration response of the six hit compounds on fibroblast proliferation as measured by the percentage of the TGFβ (DMSO) positive control. (**C**) Concentration response of the six hit compounds on fibroblast apoptosis at 48 h post-compound addition as measured by the activation of caspase 3/7 (see **Suppl. Fig. S4** for representative image). (**D**) Concentration response of the six hit compounds on scratch wound healing as measured by wound confluence at 24 h presented as a percentage of DMSO control (see **Suppl. Fig. S4** for representative image).

The induction of αSMA is integral for fibroblast to myofibroblast differentiation and is often associated with subsequent deposition of ECM. We therefore established a screening assay to simultaneously evaluate the effect of the six most potent hits from the ECM deposition assay upon αSMA expression and proliferation in primary human fibroblasts (**Fig. 4A**). The assay setup and compound treatment mirrored the ECM assay. Briefly, primary lung fibroblasts were plated subconfluently in RPTEC media in 384-well plates and treated with TGFβ (5 ng/nL) for positive control or without TGFβ for negative control. Drug treatments were added in the presence of the inflammatory stimulus TGFβ. αSMA actin induction could be observed on day 4 but was most uniform by day 7; therefore, the assay was analyzed on day 7. Briefly, plates were fixed and labeled with anti-αSMA antibody, Hoechst 33342, and HCS CellMask green and imaged on an ImageXpress (Molecular Devices) as described in Materials and Methods. The expression of αSMA was reduced by all six compounds in a concentration-dependent manner similar to the reduction in ECM deposition seen with each compound (**Fig. 4A**).

#### Primary Human Lung Fibroblast Proliferation

Primary human lung fibroblasts were dispensed into duplicate 384-well plates. One plate was fixed and labeled with Hoechst 33342 on day 1 (24 h after plating) prior to compound addition to provide a cell number baseline to compare with the cell number following 6 days of compound addition. Cell proliferation was estimated by subtracting the nuclei counts from the day 1 baseline from day 6 values and was normalized to a percentage of the TGFβ positive control. Proliferation of the fibroblasts was elevated by all the compounds at lower concentrations (below 1 µM) with the exception of camptothecin, which significantly reduced the lung fibroblast cell number in a concentration-dependent manner (**Fig. 4B**). Elevated proliferation induced by RepSox, diphenylene-iodonium chloride, GSK-650394, niclosamide, and fenretinide at lower doses agrees with the reduction in fibroblast differentiation (αSMA-positive cells), suggesting that the hit compounds likely reduce the TGFβ-induced terminal differentiation to myofibroblasts, and consequently have a higher proliferative phenotype than the TGFβ-stimulated control fibroblasts.

#### Induction of Apoptosis in Primary Lung Fibroblasts

Primary human lung fibroblasts were seeded in 384-well plates, compound treated the following day, and the induction of apoptosis was monitored over the following 48 h using a caspase 3/7 biosensor and an IncuCyte Zoom microscope. Assessment of caspase 3/7 activation over the 48 h period after compound addition showed that all but two compounds (camptothecin and niclosamide) induced little or no apoptosis over DMSO control (**Fig. 4C**). The apoptosis induced by camptothecin and niclosamide was largely seen at higher concentrations (1–10 µM), indicating that even with these compound hits there may be a potential therapeutic window where ECM deposition and αSMA expression are reduced at lower doses with minimal effect on cell death.

#### Inhibition of Scratch Wound Migration in Primary Lung Fibroblasts

Primary lung fibroblasts were seeded in 96-well plates at a density of 15,000 cells/well to create a confluent monolayer. Twenty-four hours after cell plating, the IncuCyte WoundMaker device was used to create a homogeneous scratch wound in each well of the 96-well plate. The IncuCyte Zoom was used to monitor fibroblast migration into the wound every 2 h. Images were analyzed using the IncuCyte Zoom Scratch Wound analysis module measuring the relative wound confluence over time. None of the six selected hit compounds showed a concentration-dependent change in the ability of the fibroblasts to migrate into the wound site of the scratch wound assays, with a reduction seen only at the highest concentrations (3–10 µM) (**Fig. 4D**). It is therefore likely that none of the compounds have a significant effect on primary human fibroblast cell migration.

#### Structural Similarity Ligand-Based Target Predictions (SEA Search)

**Figure 5.**
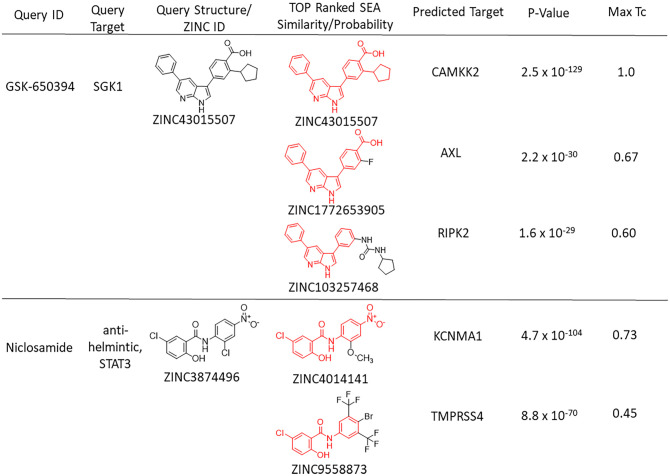
SEA search of hit compounds GSK-650394 and niclosamide to predict polypharmacology. Output compound structural similarity to query hit compound depicted in red. Prediction power indicated by probability (significant, *p* ≤ 10^−15^; highly significant, *p* ≤ 10^−40^) and Tanimoto coefficient (max Tc ≥ 0.4, 0 ≤ Tc ≤ 1.0). CAMKK2, calcium/calmodulin-dependent protein kinase kinase 2; AXL, tyrosine kinase receptor UFO; RIPK, receptor-interacting serine/threonine protein kinase; KCNMA1, calcium-activated potassium channel subunit alpha-1; TMPRSS4, transmembrane protease serine 4.

**Figure 6.**
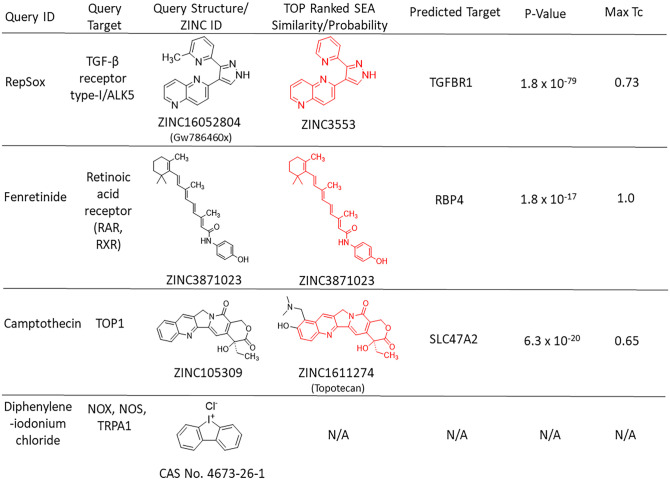
SEA search of hit compounds RepSox, fenretinide, camptothecin, and diphenylene-iodonium chloride to predict polypharmacology. Output compound structural similarity to query hit compound depicted in red. Prediction power indicated by probability (significant, *p* ≤ 10^−15^; highly significant, *p* ≤ 10^−40^) and Tanimoto coefficient (max Tc ≥ 0.4, 0 ≤ Tc ≤ 1.0). TGFBR1, TGFβ receptor type I; RBP4, retinol-binding protein 4; SLC47A2, multidrug and toxin extrusion protein 2; N/A, not applicable (no significant predictions found).

Although the six hit molecules have annotated protein targets, the promiscuous biological activity of most chemical compounds^18,19^ should be taken into account since the observed phenotype could be attributed to an unidentified target, unrelated to toxic liabilities. In order to investigate this, we performed ligand and structure based in silico search of the most potent hits. SEA search (http://sea.bkslab.org)^13^ utilizes a SMILES string to search the pharmacological space around a chemical structure to predict and rank possibly unexpected protein targets for the query molecule. The SEA output includes an output ranking the proposed target/query interaction by probability (significant, *p* ≤ 10^−15^; highly significant, *p* ≤ 10^−40^) and by similarity score to an annotated compound/drug (Tanimoto coefficient, max Tc ≥ 0.40, max Tc 1.0 = exact match).^14^ The SEA output (**Fig. 5**) for our hit compounds was potentially informative in identifying additional potential targets for our most potent hit, niclosamide (KCNMA1 and TMPRSS4), as well as GSK-650394 (CAMKK2, AXL, RIPK1). Four of the hits (RepSox, fenretinide, camptothecin, and diphenylene-iodonium chloride) did not produce significant SEA predictions beyond their known targets (**Fig. 6**).

#### Network Analysis of Hit Compounds

**Figure 7.**
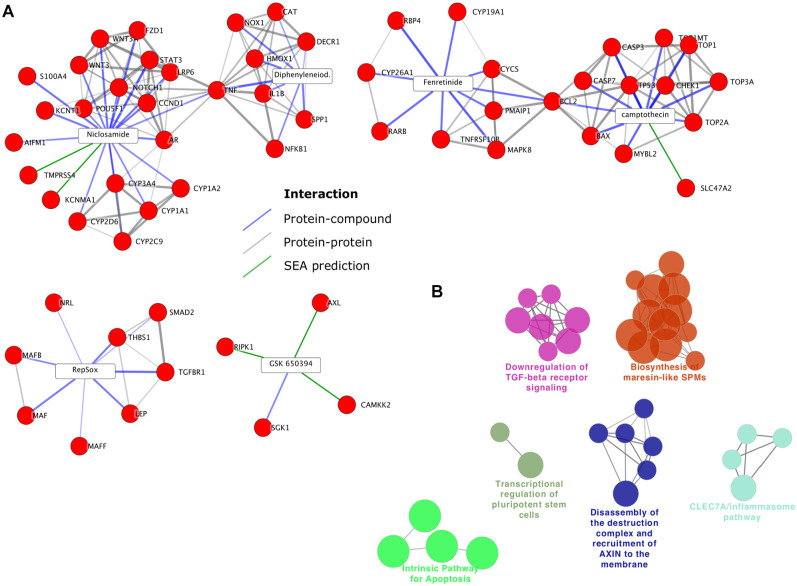
Network analysis of antifibrotic compounds. (**A**) Proteins (red circles) interacting with hit compounds (white boxes) were identified using the STITCH-STRING database of protein–protein interactions. Edge thickness denotes the STRING confidence score (thicker higher confidence). (**B**) ClueGO functional analysis of hit compound networks using Reactome pathways (*p* < 0.05, Bonferroni step-down correction). Greater node size denotes greater significance.

Network analysis using the STITCH-STRING database of protein–chemical interactions was used to identify and visualize the target protein networks of the six hit compounds (**Fig. 7A**). GSK-650394 only had STITCH-STRING interaction with its target SGK1. Functional analysis of the hit compound protein targets showed enrichment of pathways regulating maresin synthesis, TGFβ receptor signaling, WNT signaling, apoptosis, inflammasomes, and transcriptional regulation of pluripotent stem cells (**Fig. 7B**).

## Discussion

The ECM represents a reinforced composite of structural proteins (collagen, laminin, fibronectin, etc.) that are organized as fibrillar structures embedded within a viscoelastic gel that consists of proteoglycans, glycoproteins, water, growth factors, and other metabolites secreted by cells.^20^ The ECM provides both structural support and biochemical signals for multicellular tissue and organ systems. The composition and structure of the ECM is sensed by cell surface receptors such as heterodimeric integrins, cell surface glycoproteins such as dystroglycan, and CD44 and receptor tyrosine kinases such as the discoidin domain receptor family to regulate cell function.^21,22^ The immune response in the lung is fundamentally protective; however, chronic and progressive lung inflammation can be harmful. Persistent release of pro-inflammatory cytokines and chemokines elicits a number of aberrant biological responses in the lung, including chemotaxis of leukocytes, angiogenic activity, and induction of ECM synthesis and cell proliferation, which contribute to lung injury and pulmonary fibrosis.^23^ ECM deposition and altered ECM remodeling contribute to a decline in tissue elasticity and lung function, and altered ECM is considered not only a consequence but also a driver of progressive fibrosis. A spectrum of increased pulmonary fibrogenesis is observed in patients with COVID-19 from both nonventilated and mechanically ventilated patients, and in fatal cases of COVID-19, pulmonary fibrosis is generally present at autopsy.^1–3^ Recent proteomic studies indicate significant changes in the composition and expression of multiple proteins associated with ECM homeostasis in the lungs of patients with COVID-19.^24^

Altered ECM deposition and remodeling therefore appear to represent a key pathological feature of lung fibrosis and a potential therapeutic target to prevent, delay, or alleviate long-term morbidity and mortality. Traditional approaches for quantifying ECM production and turnover within in vitro assays include the incorporation and release of radiolabeled amino acids.^25,26^ However, these assays are technically challenging and not amenable to automated high-throughput screening. The development of the “Scar-in-a-Jar” model of fibrosis enabled optical image-based analysis of both fibroblast cell phenotypes and ECM deposition in each well of a 24-well assay plate.^27^ Holdsworth et al. developed this assay concept further using an in vitro model of renal epithelial cells that, following TGFβ stimulation of ECM synthesis, are lysed prior to fixation, immunostaining, and high-content image analysis of ECM deposition in 96- and 384-well plates.^10^ We further adapted this assay to monitor the deposition of the ECM proteins; collagen types I, III, and IV; and fibronectin in primary human lung fibroblasts cultured in 384-well plate formats. Following screening, multiparametric high-content analysis of ECM deposition, and hit confirmation, we identified the following six small-molecule drugs with potent activity upon inhibiting ECM deposition: RepSox, niclosamide, GSK-650394, fenretinide, camptothecin, diphenylene-iodonium chloride.

GSK-650394 is annotated as an antagonist for serum- and glucocorticoid-regulated kinase 1/2 (SGK1/2) and calcium/calmodulin-dependent kinase kinase 2 (CAMKK1/2).^28,29^ The SEA search in fact indicated a high degree of similarity and significance to multiple kinases based on its 3,5-diphenyl-1*H*-pyrrolo[2,3-b]pyridine core structure, including AXL receptor tyrosine kinase^30^ and RIPK1 (receptor-interacting serine/threonine-protein kinase 1)^31^ (**Fig. 5**). Although all the above targets are expressed in the lung (medium to high expression) (https://www.proteinatlas.org/), SGK1 overexpression has previously been implicated to play a role in inflammation resolution,^32^ pulmonary fibrosis,^33^ and fibronectin formation in other fibrotic diseases.^34^ Therefore, SGK1 would appear to be a bona fide target in our model system; however, the effect of GSK-650394 on other targets cannot be ruled out.

Niclosamide, our most potent hit on the fibronectin deposition assay (~60 nM) (**Fig. 3B**), generated some potentially interesting SEA outputs. Originally used in the treatment of tapeworm, niclosamide is also active against STAT3 at micromolar concentrations, and the JAK2/STAT3 pathway has been shown to be active in IPF.^35^ Niclosamide has also been shown to have anti-inflammatory and bronchodilation properties via TMEM16A antagonism^36^ and has been investigated for activity against SARS-COV (2007)^37^ while currently being reinvestigated for SARS-COV-2 (NCT04399356) with a view to reformulation as an aerosol for respiratory tract drug delivery.^38^ Although these latter activities refer to effects on viral replication and αSMA expression, combined with the SEA search output, they suggest that niclosamide is antagonistic against a number of targets that, potentially, all contribute to the phenotypes observed. The two most significant predictions from the SEA search for targets of niclosamide include KCNMA1 (calcium-activated potassium channel subunit alpha-1) and TMPRSS4 (transmembrane protease serine 4) (**Fig. 5**), both of which have been associated with pulmonary fibrosis. KCNMA1 is the main subunit forming the large-conductance potassium channel (BK channel), the activity of which is elevated in fibroblasts from patients with IPF and can affect the expression of αSMA by increasing intracellular calcium, required for myofibroblast differentiation leading to lung fibrosis.^39^ TMPRSS4 is similarly upregulated in IPF,^40^ and its possible inhibition by niclosamide could have an effect in vivo; however, it is not reported to be expressed in lung fibroblasts^40^ and thus is unlikely to be relevant in our cell-based model. TMPRSS4 has been linked with SARS-COV-2 cell entry and infection of several host cell types.^41,42^ Published studies and our network analysis (**Fig. 7**) indicate niclosamide also inhibits the WNT signaling pathway,^43^ and WNT signaling has previously been implicated in lung fibrosis.^44^ Therefore, the unique polypharmacology properties of niclosamide may provide added benefit to COVID-19 patients by targeting distinct mechanisms associated with SARS-COV-2 infection and lung fibrosis. Overall, the low nanomolar concentration of niclosamide required to decrease ECM deposition and αSMA expression (**Figs. 3B and 4A**) combined with relatively low toxicity and apoptosis induction at concentrations <100 nM (**Fig. 4B–D**) makes a strong case for further investigation in all stages of COVID-19.

Of the remaining hits from our screen, RepSox anti-TGFβ pathway activity is clearly an established target for lung fibrosis;^45,46^ however, clinical trials of small-molecule and biological inhibitors of TGFβ signaling have not resulted in approved therapeutics.^47^ Fenretinide, a synthetic retinoid, has mainly been investigated in an anticancer context but also has anti-inflammatory and antiviral properties.^48^ Fenretinide has been demonstrated to exhibit antifibrotic properties in hepatic fibrosis models where the mechanism of action is thought to be mediated via reactive oxygen species (ROS) generation and hepatic stellate cell apoptosis.^49^ Its drug development has been inhibited by its poor oral bioavailability requiring new formulations^50^ and, interestingly, has recently been suggested as an adjuvant for COVID-19 treatment via pulmonary delivery.^51^ Camptothecin, a well-characterized antineoplastic agent targeting DNA topoisommerase I, has been reported as modulating HIF1α activity and hence inflammation,^52^ implying a potential antifibrotic role. Camptothecin has also been previously shown to inhibit collagen I deposition in activated dermal fibroblasts.^53^ However, camptothecin itself has been superseded in the clinic by irinotecan and topotecan, and both compounds were inactive in our ECM deposition readouts, so the relevance of this drug class in lung fibrosis remains to be determined. Finally, diphenylene-iodonium chloride is a seemingly unremarkable, hypervalent organoiodine compound and is the least active of our validated hits (EC_50_ 4.2 µM fibronectin deposition) (**Fig. 3B**). Nevertheless, it has been reported as attenuating IPF-associated senescent lung fibroblasts and TGFβ-induced peroxide formation via inhibition of NADPH oxidases (NOX).^54,55^ More specifically, diphenylene-iodonium chloride has been shown to protect from bleomycin-induced lung fibrosis in vivo, quantified in part by reduction of αSMA-expressing myofibroblasts, through inhibition of NOX4.^56^ The activity of diphenylene-iodonium chloride includes flavoenzymes in general,^57^ as well as a broad-spectrum bacteriocide.^58^ It is currently in unrelated clinical trials for motion sickness, formulated as a nasal gel (DPI-386, NCT04184115, phase 3), but is otherwise an unapproved drug and any utility in COVID-19-related fibrosis remains to be determined. Protein interaction network analysis performed on our confirmed hit compounds has identified several other pathway and protein targets potentially implicated in the pharmacological responses observed in our phenotypic lung assays, including maresin synthesis, WNT signaling, apoptosis, inflammasomes, and stem cell transcriptional regulation (**Fig. 7**). Mapping of these pathways to drug target databases and further experimental investigation may reveal additional drug repurposing opportunities in lung fibrosis.

There are only two drugs approved for specifically treating IPF, pirfenidone and nintedanib. These have proven partially effective at controlling lung diseases such as IPF, and studies are ongoing as to their effectiveness toward the fibroproliferative response in COVID-19.^1^ However, standard oral administration of pirfenidone and nintedanib would be unsuitable for patients on a ventilator, and thus other formulations and administration routes would be required. The use of pirfenidone and nintedanib for effective treatment of lung fibrosis is also limited by their potency and selectivity; while they have both been proven effective in reducing functional decline and disease progression in IPF, neither represents a cure, and most patients continue to progress despite treatment. Pirfenidone was present in LOPAC and was not classed as a hit at the 1 µM concentration tested in our screen. This was not surprising, as previous studies have reported in vitro activity of pirfenidone in the millimolar range.^59,60^ Nintedanib was not present in our screening library; it is a multitarget kinase inhibitor and has previously been implicated in perturbing multiple cellular functions, including ECM deposition, cell proliferation, migration, and apoptosis.^61–64^ It therefore remains to be determined if nintedanib would be classed as a hit in our screen using our criteria of selective inhibition of ECM deposition independent of overt cell death. However, the purpose of our high-content lung fibroblast ECM deposition screen and suite of secondary assays is to identify potent and selective drug repurposing opportunities and new drug discovery programs that provide improvements over the existing agents currently used for IPF.

Guided by the phenotypic screening “rule of 3”—(1) relevant/primary human cell type, (2) disease-relevant stimulus, and (3) assay readout proximity to the clinical endpoint^65^—we describe a high-throughput lung fibroblast ECM deposition assay in 384-well plate format and a cascade of secondary lung fibroblast phenotypic assays to support the discovery of new therapies for tackling lung fibrosis. The foundation of the assay system is cryopreserved primary human lung fibroblasts subjected to limited in vitro cell culture passage. The primary human lung ECM deposition assay uses TGFβ as a pro-fibrotic inflammatory stimulus to induce ECM deposition from lung fibroblasts. TGFβ has previously been identified as an important pro-inflammatory agent in fibrosis and has been proposed as a potential therapeutic target in COVID-19.^45,46,66^ The primary phenotypic screening assay endpoint for hit selection includes multiparametric analysis of the deposition of multiple ECM proteins associated with clinical lung fibrosis, including collagens I, III, and IV and fibronectin. A significant challenge presented by our cell-free high-content ECM deposition assay endpoint is triaging cytotoxic compounds; thus, our approach monitors cytotoxicity in the same assay wells as those used for the final ECM assay endpoint. In the current assay format, cytotoxicity was assessed in live cell images using a semiquantitative method of cell confluence to highlight potential cytotoxic effects for visual inspection and confirmation. This assessment of cytotoxicity in live cell assay plates could be improved with more automated label-free analysis of cell health using artificial intelligence and machine learning-based classification methods.^67,68^

In this pilot study, our primary screen and secondary assays demonstrate high levels of reproducibility and signal to noise and have identified highly potent drugs that may have potential for drug repurposing opportunities. Our primary human lung ECM deposition assay and suite of secondary assays are suitable for high-throughput screening of larger diverse chemical libraries and siRNA or CRISPR genetic screens. Therefore, the assay protocols, image analysis, and pathway network analysis tools described in our study can be utilized in further small-molecule and function genomic phenotypic screens to identify novel chemical starting points and/or therapeutic targets to advance drug discovery in lung fibrosis.

## Supplemental Material

sj-pdf-1-jbx-10.1177_24725552211019405 – Supplemental material for Application of a High-Content Screening Assay Utilizing Primary Human Lung Fibroblasts to Identify Antifibrotic Drugs for Rapid Repurposing in COVID-19 Patients Click here for additional data file. Supplemental material, sj-pdf-1-jbx-10.1177_24725552211019405 for Application of a High-Content Screening Assay Utilizing Primary Human Lung Fibroblasts to Identify Antifibrotic Drugs for Rapid Repurposing in COVID-19 Patients by John A. Marwick, Richard J. R. Elliott, James Longden, Ashraff Makda, Nik Hirani, Kevin Dhaliwal, John C. Dawson and Neil O. Carragher in SLAS Discovery
