# Supplementary material for: Application of a High-Content Screening Assay Utilizing Primary Human Lung Fibroblasts to Identify Antifibrotic Drugs for Rapid Repurposing in COVID-19 Patients
Source: SLAS Discov. 2021 Jun 2;26(9):1091–106. doi: 10.1177/24725552211019405 (PMC8458684; doi:10.1177/24725552211019405)

SUPPLEMENTARY FIGURES 1-4

Application of a high-content screening assay utilising primary human lung fibroblasts to identify potential antifibrotic drugs for rapid clinical deployment in COVID-19 patients

John A. Marwick [1,2], Richard J.R. Elliott[1], James Longden[3], Ashraff Makda[1], Nik Hirani[2], Kevin Dhaliwal[2], John C. Dawson[1] and Neil O. Carragher[1]*

[1]Cancer Research UK Edinburgh Centre, MRC Institute for Genetic and Molecular Medicine, University of Edinburgh, UK

[2]Centre for Inflammation Research, Queens Medical Research Institute, University of Edinburgh, UK

[3]Center for Clinical Brain Sciences, Chancellors Building, University of Edinburgh, UK

*** Correspondence:** n.carragher@ed.ac.uk

Supplemental Figures

Supplementary Fig. 1. ECM deposition and cell health (RepSox and Niclosamide). Concentration responses of repsox (top) and niclosamide (bottom) on fibronectin, collagen I+III and collagen IV deposition with representative live cell images for compound cytotoxicity. [Conc.: concentration].

Supplementary Fig. 2. ECM deposition and cell health (Camptothecine and Fenretinide (Retinoic Acid p-nitroanilide)). Concentration responses of campothecine (top) and fenretinide (bottom) on fibronectin, collagen I+III and collagen IV deposition with representative live cell images for compound cytotoxicity. [Conc.: concentration].

Supplementary Fig. 3. ECM deposition and cell health (GSK-650394 and Diphenyleneiodonium Chloride). Concentration responses of GSK-650394 (top) and diphenyleneiodonium chloride (bottom) on fibronectin, collagen I+III and collagen IV deposition with representative live cell images for compound cytotoxicity. [Conc.: concentration; Cl: chloride].

Supplementary Fig. 4. Suite of secondary lung phenotypic assays. (**A**) Representative high-content image from the αSMA expression (fibroblast to myofibroblast differentiation) and cell count assay; αSMA (red), Hoechst 33342 (blue), CellMask Green (green). (**B**) Representative live cell image from the apoptosis assay taken at 48 hours post compound addition (activation of caspase 3/7); active caspase 3/7 (green). (**C**) Representative live cell image from the scratch wound assay taken at 0 hours post compound addition.

Supplementary Fig S1: ECM deposition and cell heath (RepSox and Niclosamide)

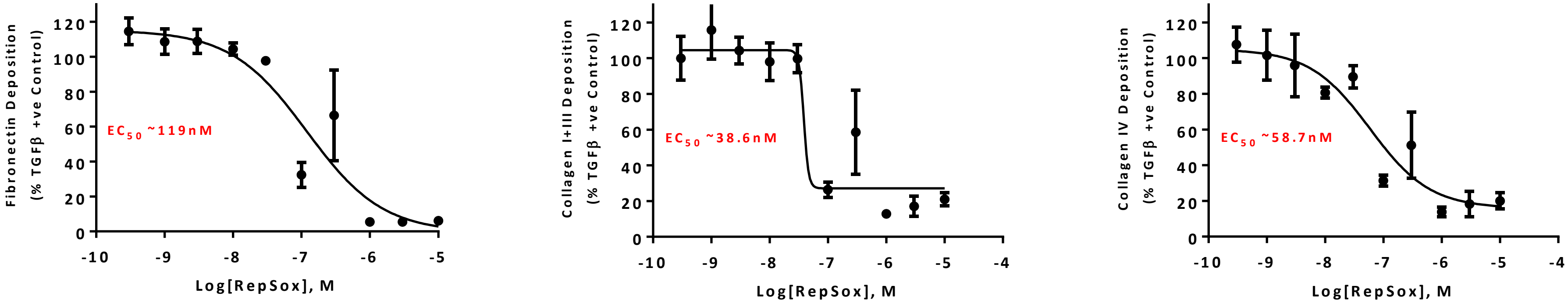

Conc. (nM)	Incucyte
10000	Alive
3000	Alive
1000	Alive
300	Alive
100	Alive
30	Alive
10	Alive
3	Alive
1	Alive
0.3	Alive

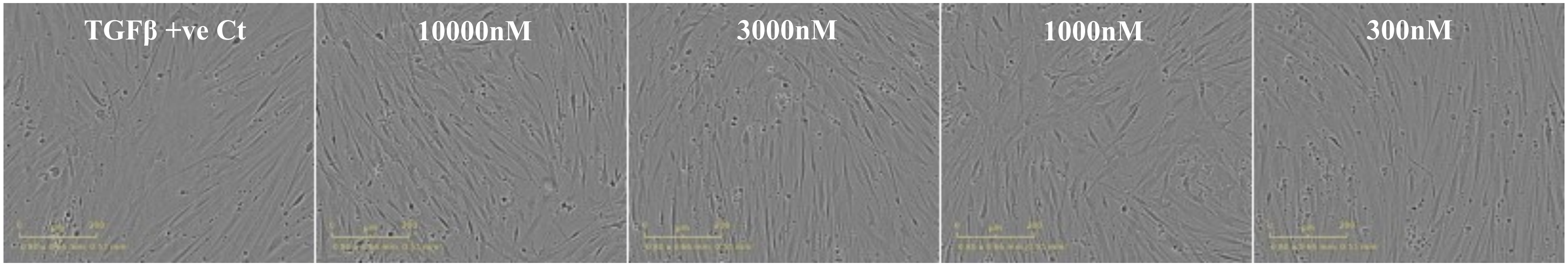

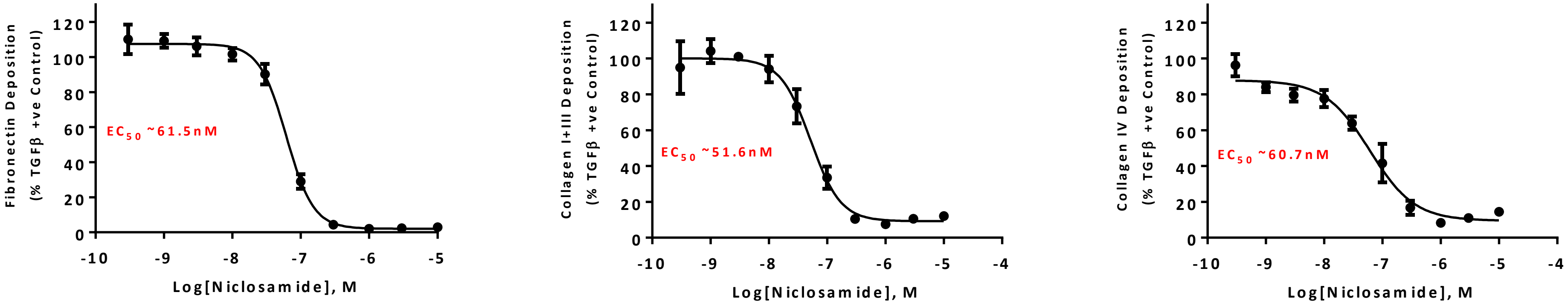

Conc. (nM)	Incucyte
10000	Dead
3000	Dead
1000	Dead
300	Dying
100	Alive
30	Alive
10	Alive
3	Alive
1	Alive
0.3	Alive

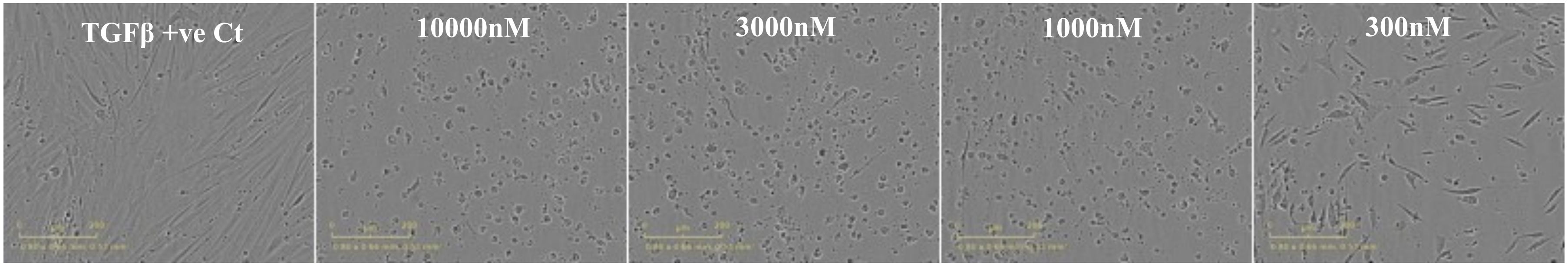

Supplementary Fig S2: ECM deposition and cell heath (Camptothecin and Fenretinide)

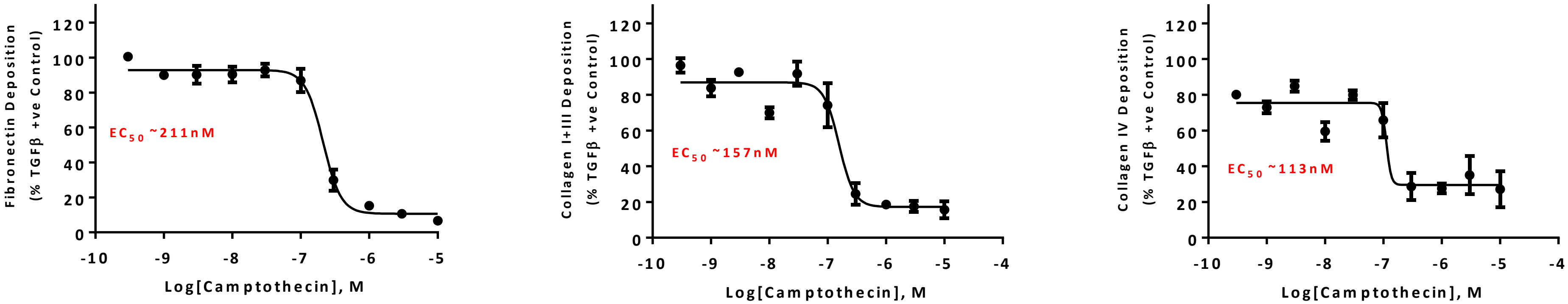

Conc. (nM)	Incucyte
10000	Dead
3000	Dying
1000	Dying
300	Alive
100	Alive
30	Alive
10	Alive
3	Alive
1	Alive
0.3	Alive

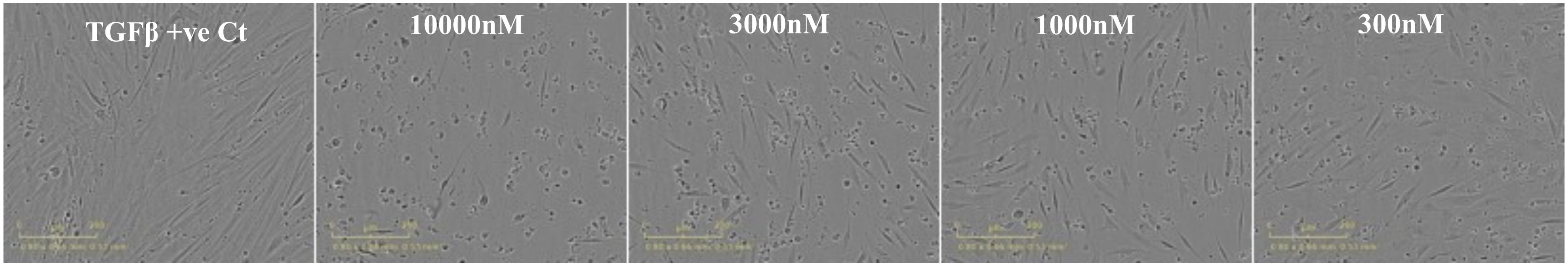

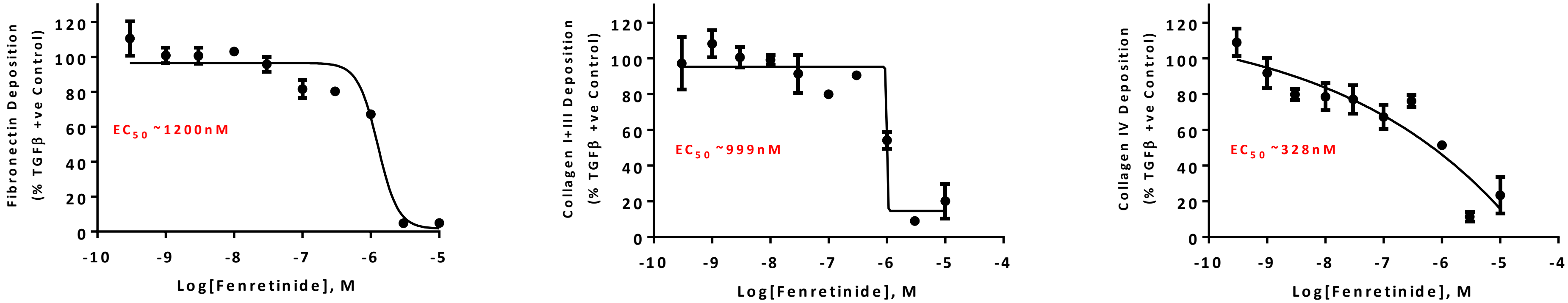

Conc. (nM)	Incucyte
10000	Dead
3000	Dying
1000	Alive
300	Alive
100	Alive
30	Alive
10	Alive
3	Alive
1	Alive
0.3	Alive

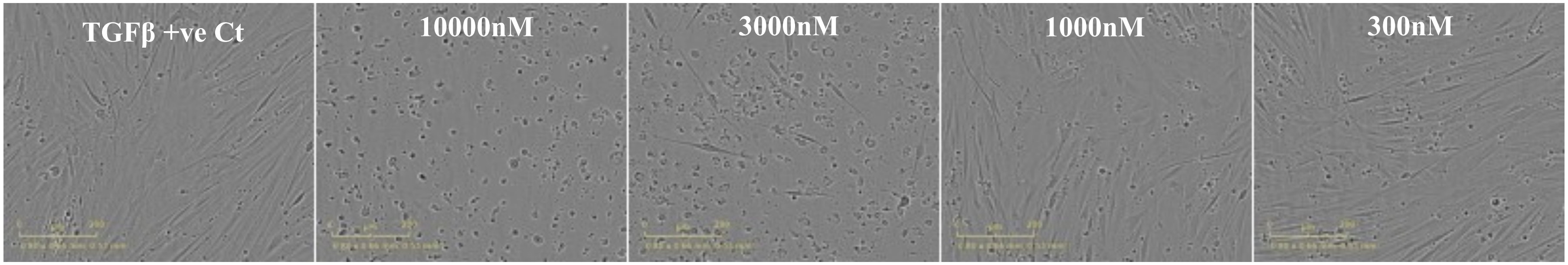

Supplementary Fig S3: ECM deposition and cell heath (GSK-650394 and Diphenyleneiodonium Chloride)

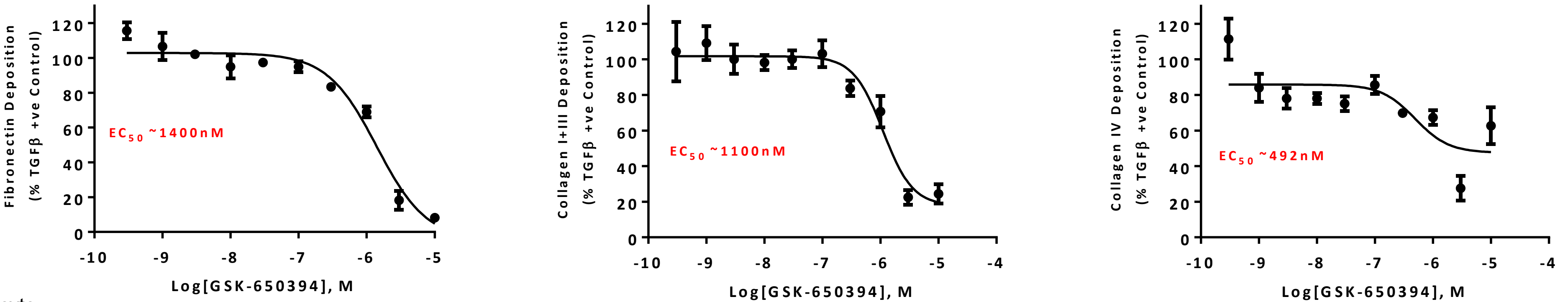

Conc. (nM)	Incucyte
10000	Dying
3000	Dying
1000	Alive
300	Alive
100	Alive
30	Alive
10	Alive
3	Alive
1	Alive
0.3	Alive

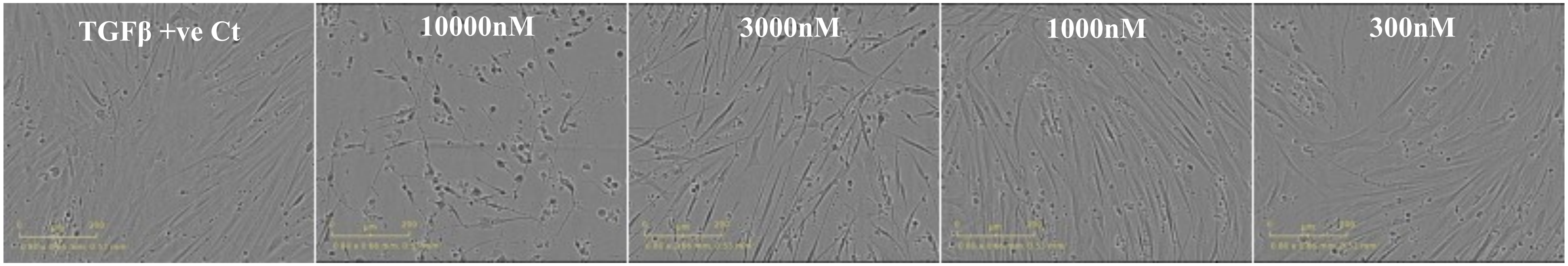

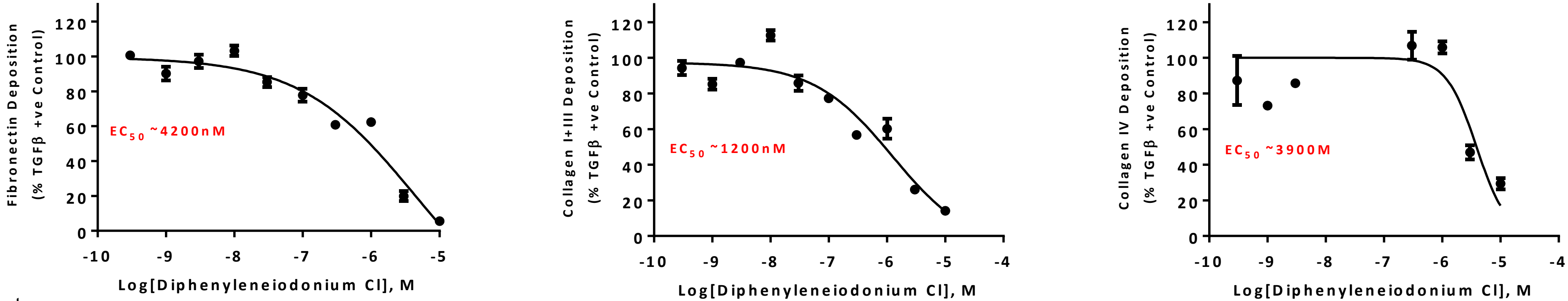

Conc. (nM)	Incucyte
10000	Dead
3000	Dying
1000	Alive
300	Alive
100	Alive
30	Alive
10	Alive
3	Alive
1	Alive
0.3	Alive

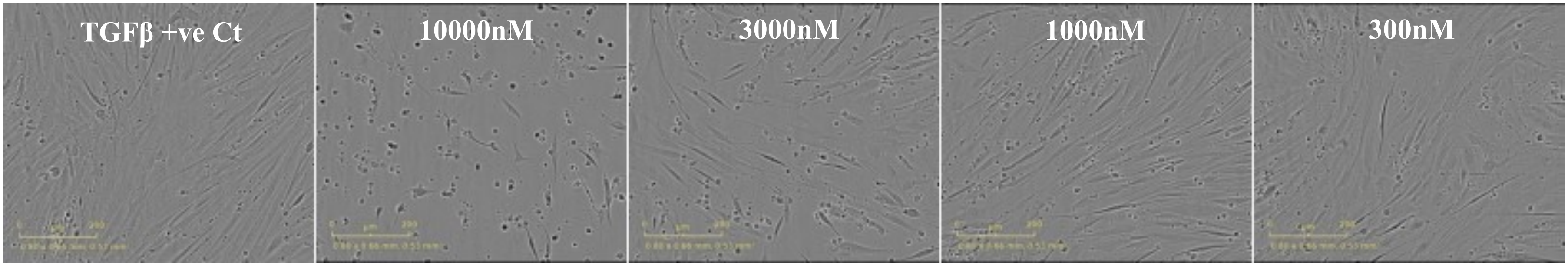

Supplementary Fig S4: Suite of secondary lung fibroblast phenotypic assays:

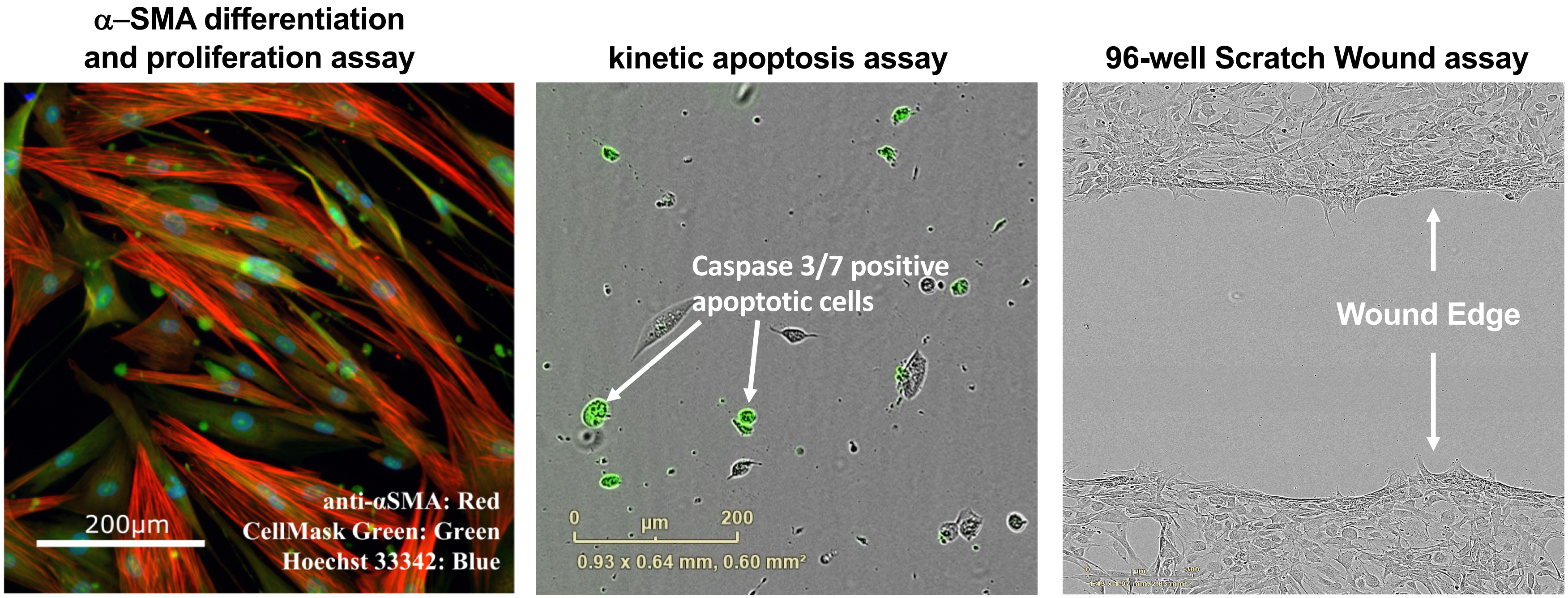

Supplement: sj-pdf-1-jbx-10.1177_24725552211019405 – Supplemental material for Application of a High-Content Screening Assay Utilizing Primary Human Lung Fibroblasts to Identify Antifibrotic Drugs for Rapid Repurposing in COVID-19 Patients [file sj-pdf-1-jbx-10.1177_24725552211019405.pdf]
